# Relationship between Glutathione-Dependent Enzymes and the Immunohistochemical Profile of Glial Neoplasms

**DOI:** 10.3390/biomedicines10102393

**Published:** 2022-09-25

**Authors:** Larisa Obukhova, Tatiana Kopytova, Elena Murach, Natalya Shchelchkova, Claudia Kontorshchikova, Igor Medyanik, Natalia Orlinskaya, Artem Grishin, Michael Kontorshchikov, Dariya Badanina

**Affiliations:** Federal State Budgetary Educational Institution of Higher Education, The “Privolzhsky Research Medical University” of the Ministry of Health of the Russian Federation, 603005 Nizhny Novgorod, Russia

**Keywords:** glutathione, glutathione reductase, glutathione transferase, glutathione peroxidase, gliomal molecular genetic markers, IDH1, MGMT, Ki67, p53

## Abstract

This research aimed to investigate the relationships between the parameters of glutathione metabolism and the immunohistochemical characteristics of glial tumors. Postoperative material from 20 patients with gliomas of different grades of anaplasia was analyzed. Bioinformatic analysis of the interactions between the gliomas’ immunohistochemical markers and their glutathione-dependent enzymes was carried out using the STRING, BioGrid, while Signor databases revealed interactions between such glioma markers as IDH and p53 and the glutathione exchange enzymes (glutathione peroxidase, glutathione reductase, glutathione S-transferase). The most pronounced relationship with glutathione metabolism was demonstrated by the level of the nuclear protein Ki67 as a marker of proliferative activity, and the presence of the IDH1 mutation as one of the key genetic events of gliomagenesis. The glutathione system is an active participant in the body’s antioxidant defense, involving the p53 markers and MGMT promoter methylation. It allows characterization of the gliomal cells’ status at different stages of tumor development.

## 1. Introduction

The glutathione system plays an important role in protecting cells from free-radical oxidation. Indicators characterizing glutathione system functionality include the concentration of oxidized (GSSG) and reduced (GSH) glutathione, the content and activities of glutathione peroxidase (GSP), glutathione reductase (GSR), and glutathione transferase (GGT) [1]. It has been revealed [2,3] that changes of reduced glutathione levels can be observed in various human cancer cells and are an important factor in the development of cancer pathology.

The most commonly used markers determined by immunohistochemistry in neuro-oncology include IDH mutations, MGMT promoter methylation, p53 protein, and the level of Ki67 controlling proliferative activity [4].

The IDH gene encodes the tricarboxylic acid cycle enzyme, isocitrate dehydrogenase [5]. This enzyme is known to be located in the cytoplasm. Its main function is to catalyze decarboxylation of isocitrate. As a result of these reactions, alpha-ketoglutarate and NADPH2 (reduced form) are formed. It was found [6] that IDH mutations are involved in glyomagenesis at the earliest stages of tumor growth.

Tamura (2012) found that tumor suppressor p53 induces apoptosis during genome damage [7]. This blocks the cell division cycle preventing the accumulation of genetically defective cells. In turn, TR53 mutations inactivate the relevant protein synthesis causing oncogenesis development [8].

Nuclear protein Ki67 is involved in ribosomal RNA synthesis [9]. The protein is undetectable in the resting phase, while its expression can reflect tissue proliferation activity. As a result, Ki67 is recommended as an auxiliary marker for differential diagnosis of the following gliomal stages: Low Grade (I, II) and High Grade (III, IV) [10,11]

Enzyme o-6-methylguanine-DNA methyltransferase is known to be involved in DNA repair [12]. As the methyl group attached to the promoter prevents gene expression [13], MGMT methylation can be used as a predictive marker of tumor response to chemotherapy involving the application of alkylating agents [14].

This study aims to find relationships between glutathione metabolism parameters and gliomal molecular genetic markers (IDH1; MGMT; Ki67; p53).

## 2. Materials and Methods

### 2.1. Materials

Tissues from tumors, the peritumoral zone and adjacent noncancerous regions were collected as postoperative material before anti-tumor therapy (with informed consent) at the University Hospital of the Federal State Budgetary Educational Institution of Higher Education, the “Privolzhsky Research Medical University” of the Ministry of Health of the Russian Federation. The histological diagnosis was established according to the WHO classification of CNS tumors [4]. The detailed information on patients is listed in Additional file 1: Table S1.

### 2.2. Measurement of Tumor Markers

Immunohistochemical study is the basic method of determining glioma markers: Ki67, IDH mutation, and p53 protein. MGMT promoter methylation may also be tested by immunohistochemical methods, but their sensitivity will be limited in some cases. Despite the complexity of assessing immunohistochemical methods, they are much more widely used in the in-patient environment compared to molecular genetics studies.

Besides, immunohistochemistry enables accurate assessment and visual representation of quantitative parameters of markers, provided that the tumor substrate volume is sufficient, and helps to demarcate the tumor tissue from the peritumoral zone.

The fragments were considered to have a sufficient volume of material if they might be used to prepare slices with an area of 100–200 mm^2^ and a thickness of 20 μm in case of material cellularity equaling to at least 70%.

**Figure 1:**
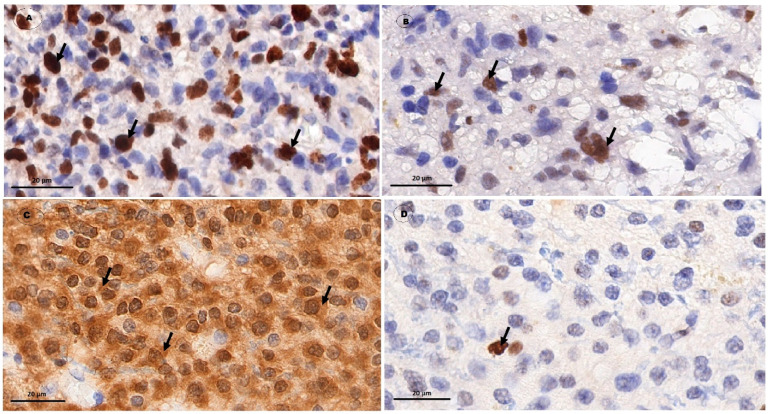
Immunohistochemical study of glial tumor markers. Magnification ×1000, Bar: 20 µm. (**A**) Level of nuclear non-histone protein, a marker of Ki67 cell proliferation in Grade IV glioblastoma material. The pronounced, diffuse, brown nuclear staining (arrows) shows an antigen-antibody binding reaction (up to 50% of cells in hot spots), indicating a high Ki67 proliferation index. (**B**) Presence of p53 marker in Grade III anaplastic astrocytoma material. The moderately pronounced, diffuse nuclear staining (arrows) shows an antigen-antibody binding reaction, indicating the presence of p53 mutant protein. (**C**) Presence of IDH1 mutation in Grade II diffuse astrocytoma material. The cytoplasm with moderately pronounced, diffuse, brown staining shows an antigen-antibody binding reaction (arrows), indicating the presence of an IDH1 mutation. (**D**) Presence of MGMT methylation in Grade II diffuse astrocytoma material. Pointwise (less than 10% of cells) the brown nuclear staining (arrow) shows an antigen-antibody binding reaction, indicating the presence of MGMT promoter methylation.

Postoperative material was fixed in 10% formalin solution and was processed according to the standard procedure. The following antibody clones were used: Anti-IDH1 R132H (Dianova International, Castelldefels, Spain); Anti-MGMT (clone EP337), item AC-0307RUO (Epitomics, Burlingame, CA, USA), Anti-p53 (clone DO-7) (Leica biosystems, Heidelberg, Germany), and Ki67 antibodies (clone SP6) (Thermo Scientific, Waltham, MA, USA). The presence of the IDH1 mutation was assessed by the presence of its cytoplasmic expression (Figure 1). The levels of MGMT, Ki67, and p53 markers were determined by the proportion of positive nuclear staining (Figure 1). The values obtained were expressed as the proportion of stained cells in 10 fields of view at ×400 magnification [15,16]. When investigating the presence of MGMT promoter methylation, nuclear staining in less than 15% of cells was considered positive [17].

### 2.3. Preparation of Tissue Homogenates for Biochemical Research

The tissue homogenates were obtained in a refrigerated room at 0 °C. For this purpose, postoperative material was washed in 0.32 M, pH = 7.4 sucrose solution, and all membranes were removed. The tissue was then homogenized at 200 rpm in a homogenizer (glass-Teflon) in a 10-fold volume of release medium containing 0.32 M sucrose, 10 mM tris-HCl, and 1 mM EDTA, pH = 7.4.

### 2.4. Analysis of Glutathione Metabolism Parameters

The content of oxidized and reduced glutathione in tissue homogenates was determined by colorimetry according to the protocol for the Quantification kit for oxidized and reduced glutathione (Cat. No. 38185, Sigma-Aldrish, Darmstadt, Germany). Absorption was measured at 412 nm using an EPOCH tablet spectrophotometer (BIO-TEK, USA).

The content of glutathione system enzymes in brain tissue homogenates was studied using the “sandwich” type immunoenzymatic method. For determining the amount of glutathione S transferase alpha 1 (GSTa1) we used a SEA609Hu ELISA Kit for Glutathione S Transferase Alpha 1, (Cloud-Clone Corp. (PRC)), for glutathione reductase (GR), a SEB314Hu ELISA Kit for Glutathione Reductase, (Cloud-Clone Corp. (PRC)), for glutathione peroxidase 1 (GPX1), a SEA295Hu ELISA Kit for Glutathione Peroxidase 1, (Cloud-Clone Corp (PRC)). The optical density was recorded at 450 nm using an EPOCH tablet spectrophotometer (BIO-TEK, USA).

The level of each enzyme was then expressed per 1 g of protein as determined by the Lowry method, using a set of reagents from Firma Syntakon LLC, (Russia).

### 2.5. Analysis of Protein-Protein Interactions

Protein-protein interactions between gliomas’ immunohistochemical markers and glutathione metabolism enzymes were analyzed using data from BioGrid (BioGRID|Database of Protein, Chemical, and Genetic Interactions (thebiogrid.org)), Signor (SIGNOR 2.0 (uniroma2.it)), SignaLink (SignaLink 3.0. (signalink.org)) and STRING (STRING: functional protein association network (string-db.org).

An integrative diagram of the resulting protein-protein interactions was developed using the “yEd Graph Editor” program (v. 3.22., yWorks GmbH, Tübingen, Germany).

### 2.6. Statistical Analysis

Data were processed statistically using the AnalystSoft Inc. package, StatPlus, version 6, Alexandria, VA, USA (www.analystsoft.com/ru/ (accessed on 28 June 2022)). The results were presented as median, quartiles of percentiles (25%; 75%). The significance of the resulting differences was assessed using nonparametric criteria (Mann-Whitney U-test). Spearman’s coefficient was also calculated for the correlation analysis.

## 3. Results

### 3.1. The Level of Molecular Genetic Markers of Gliomas with Varying Degree of Anaplasia

The immunohistochemical profile was studied in Grade I pilocytic astrocytomas, in astrocytomas and in oligodendrogliomas with anaplasia levels II and III, as well as in primary glioblastomas, midline gliomas, and Grade IV astrocytomas (Table 1).

The Ki67 proliferative index value increased as the anaplasia grade increased. The value was highest in the Grade IV gliomal group. Detection of p53 protein was not clearly correlated with the malignancy grade (Table 1). However, no p53 tumor marker was found in any of the four cases of oligodendrogliomas, although it was present in most astrocytic masses. The IDH1 mutation was seen in all cases of oligodendrogliomas and in the Grade II–IV astrocytomas. No IDH1 mutation was detected in primary glioblastomas, midline gliomas or pilocytic astrocytomas. MGMT promoter methylation was predominant in Grade I, II and III gliomas. The results are consistent with the WHO classification of primary CNS tumors 2021 [4].

### 3.2. Protein-Protein Interactions between Immunohistochemical Markers of Gliomas and Glutathione Metabolism Enzymes

**Figure 2:**
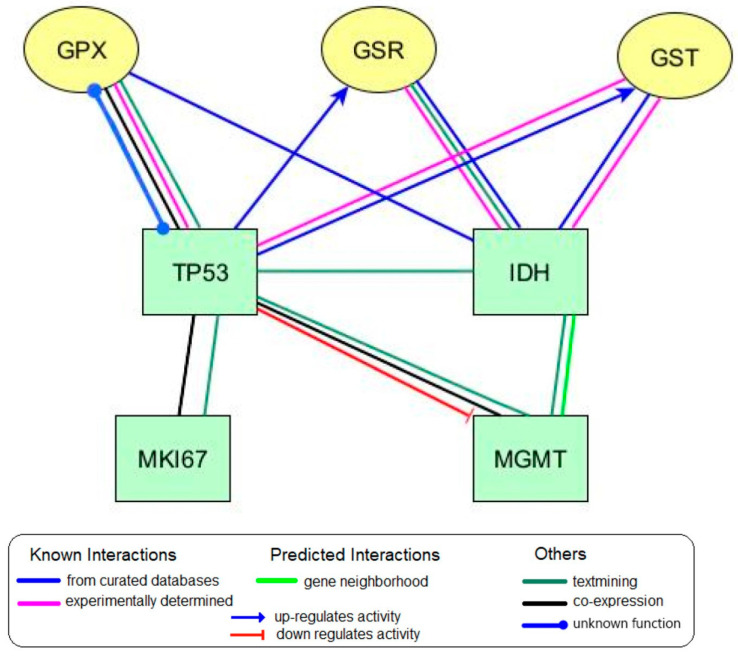
Integrated diagram of protein-protein interactions of immunohistochemical glioma oncomarkers with glutathione metabolism enzymes as per BioGrid, STRING, Signor, and SignaLink databases, using yEd Graph Editor software.

To establish the existing relationships between immunohistochemical markers of gliomas and the investigated glutathione metabolism enzymes, a bioinformatic analysis was run using databases of molecular-biological interactions. This revealed interactions between glioma tumor growth markers like IDH/TP53 and the glutathione metabolism enzymes (glutathione peroxidase (GPX), glutathione reductase (GSR) and glutathione S-transferase (GST) (Figure 2)). These data were obtained using STRING and SignaLink with 0.400 mean confidence. Protein-protein interactions were only considered if the Combined Score between the nodes was ≥0.6. Both direct and mediated effects of some proteins on the functional state of other proteins were detected.

Legend: IDH—isocitrate dehydrogenase; MGMT—O-6-methylguanine-DNA methyltransferase; MKI-67—marker of proliferation Ki67; TP53—tumor protein p53; GPX—glutathione peroxidase; GST—glutathione S-transferase; GSR—glutathione reductase

Using SignaLink, it was found that p53 affects the amounts of GST and GSR. Signor showed that p53 downregulates MGMT expression.

Using the BioGrid database, a relationship was established between the IDH1 marker with a high throughput and GSR (high throughput = 1.74).

SignaLink revealed reciprocal interactions between GPX, p53, and the IDH proteins. The local clustering coefficient between these proteins was 0.705.

Biologically relevant interactions of IDH1 and p53 with glutathione peroxidase, glutathione reductase and glutathione S-transferase were identified using protein-protein interactions databases.

### 3.3. Metabolic Parameters of Glutathione and Reactive Oxygen Species Production Depending on the Glial Tumor Zone

The levels of oxidized and reduced glutathione, glutathione peroxidase, glutathione reductase, and glutathione transferase were assessed in tumor tissues, the peritumoral zone, and their adjacent noncancerous tissues (Table 2).

The level of oxidized glutathione was significantly reduced compared to that in the adjacent noncancerous tissues both in the peritumoral zone and in the tumor tissue at all anaplasia grades, except for the tumor tissue at Grade II (Table 2).

Values of reduced glutathione were significantly elevated compared to the adjacent noncancerous tissues only in Grade II tumor tissue but decreased in both the peritumoral zone and in tumor tissues at Grades III and IV (Table 2).

The amount of glutathione peroxidase was elevated in the peritumoral zone at Grades I and II but was significantly lower than in the adjacent noncancerous tissues for Grade IV (Table 2). The level of this enzyme in the tumor tissue was high at Grade I but low at all other anaplasia grades.

The glutathione reductase level was significantly higher in the peritumoral zone at Grade II compared with the adjacent noncancerous tissues but was lower at Grades III and IV. The content of this enzyme in tumor tissue was observed to decrease as the degree of anaplasia increased (Table 2).

The amount of glutathione transferase in both the peritumoral zone and tumor tissues was higher than in the adjacent noncancerous tissues at Grades I and II. The level of this enzyme decreased at the higher grades (Table 2).

### 3.4. Characterization of Glutathione Metabolism as a Function of the Immunohistochemical Profile of Gliomas

Correlations between the glutathione metabolism enzymes and the markers of gliomal tumor growth are represented in Table 3. The greatest number of significant relationships was found for Ki67 and for MGMT.

Further, the data on glutathione metabolism parameters were split into groups depending on the relevant marker’s immunohistochemical profile. The figures show only data with significant differences between the groups.

**Figure 3:**
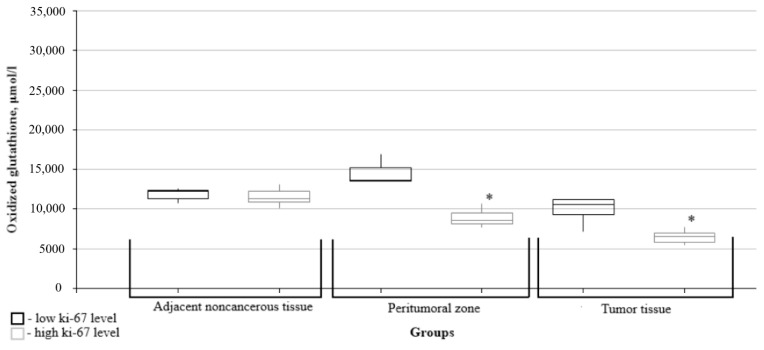
Medians and interquartile ranges (IQRs) of oxidized glutathione levels in gliomas as a function of the Ki67 mitotic index value. There are significant differences between the oxidized glutathione levels in the peritumoral zone tissues (*—Mann-Whitney U-test *p* = 0.028) and the tumors (*—Mann-Whitney U-test *p* = 0.007) for the low and high mitotic index Ki67 groups.

**Figure 4:**
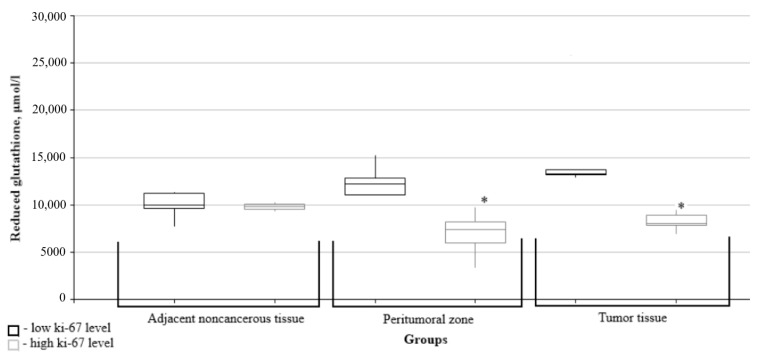
Medians and interquartile ranges (IQRs) of reduced glutathione level in gliomas as a function of the Ki67 mitotic index value. There are significant differences between the reduced glutathione levels in peritumoral zone tissues (*—Mann-Whitney U-test *p* = 0.048) and the tumors (*—Mann-Whitney U-test *p* = 0.004) for the low and high mitotic index Ki67 groups.

The levels of oxidized and reduced glutathione in the peritumoral zone and in the tumor tissues were significantly lower in the high Ki67 mitotic index group than in the low Ki67 group (Figure 3 and Figure 4).

**Figure 5:**
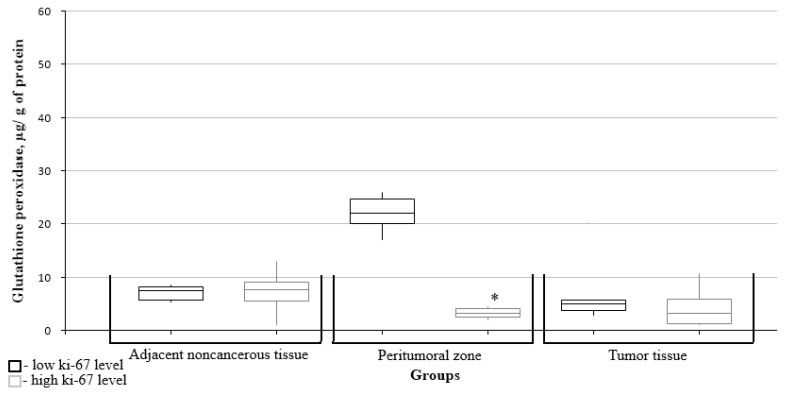
Medians and interquartile ranges (IQRs) of glutathione peroxidase levels in gliomas as a function of the Ki67 mitotic index value. There are significant differences between the glutathione peroxidase levels in peritumoral zone tissues (*—Mann-Whitney U-test *p* = 0.004) and the low and high mitotic index Ki67 groups.

The glutathione peroxidase level in the peritumoral zone was significantly lower in the high Ki67 mitotic index group (Figure 5).

**Figure 6:**
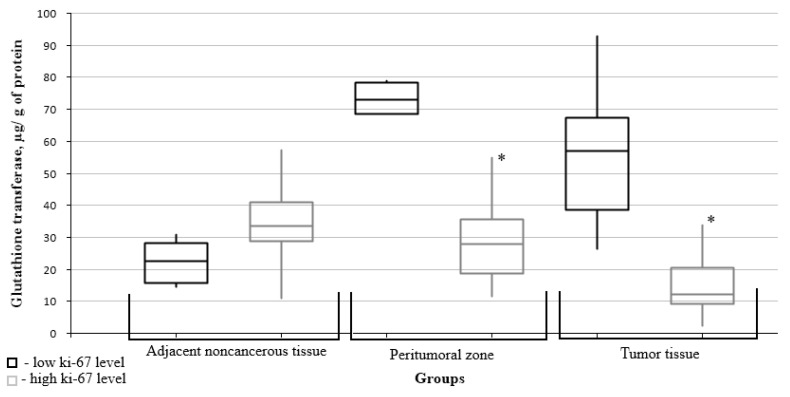
Medians and interquartile ranges (IQRs) of glutathione S-transferase levels in gliomas as a function of the Ki67 mitotic index value. There are significant differences between the glutathione transferase levels in the peritumoral zone tissues (*—Mann-Whitney U-test *p* = 0.007) and the tumors (*—Mann-Whitney U-test *p* = 0.010) for the low and high mitotic index Ki67 groups.

The glutathione transferase level in the peritumoral zone was significantly lower in the group of patients with gliomas having a high Ki67 mitotic index (Figure 6).

**Figure 7:**
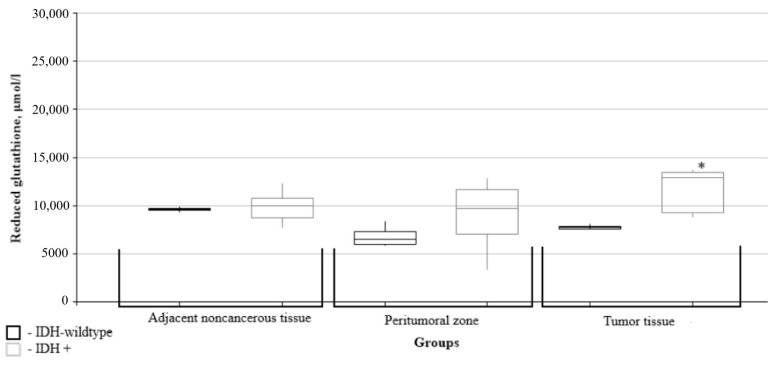
Medians and interquartile ranges (IQRs) of reduced glutathione levels in glial tumor tissues as a function of the IDH1 isocitrate dehydrogenase isoenzyme gene mutation. There are significant differences between the tumor tissue reduced glutathione levels (*—Mann-Whitney U-test *p* = 0.011) for groups with Wild type IDH1 and those expressing the IDH1 mutation.

The content of reduced glutathione in the tumor tissues of patients with gliomas and the IDH1 mutation was significantly higher than in the wild-type IDH1 group (Figure 7).

**Figure 8:**
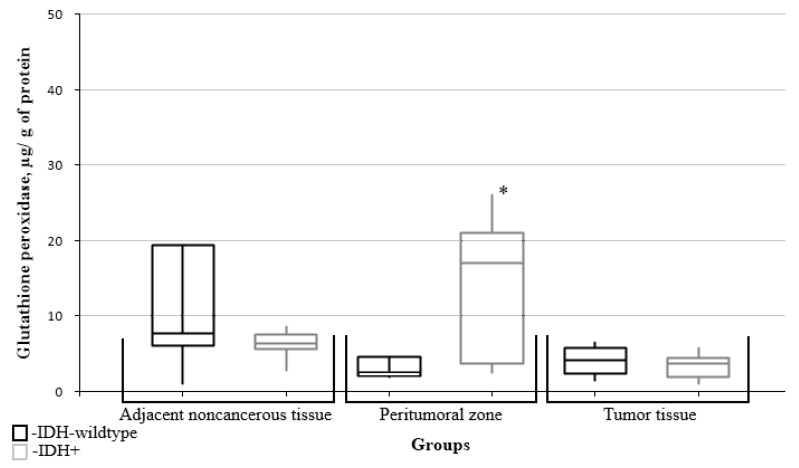
Medians and interquartile ranges (IQRs) of glutathione peroxidase levels in glial tumor tissue as a function of the IDH1 isocitrate dehydrogenase isoenzyme gene mutation. There are significant differences of the glutathione peroxidase levels between the peritumoral zone tissues (*—Mann-Whitney U-test *p* = 0.043) and the groups with Wild type IDH1 and those expressing the IDH1 mutation.

The levels of glutathione peroxidase in the peritumoral zone tissue of patients with gliomas and the IDH1 mutation were significantly higher than in the wild-type IDH1 group (Figure 8).

**Figure 9:**
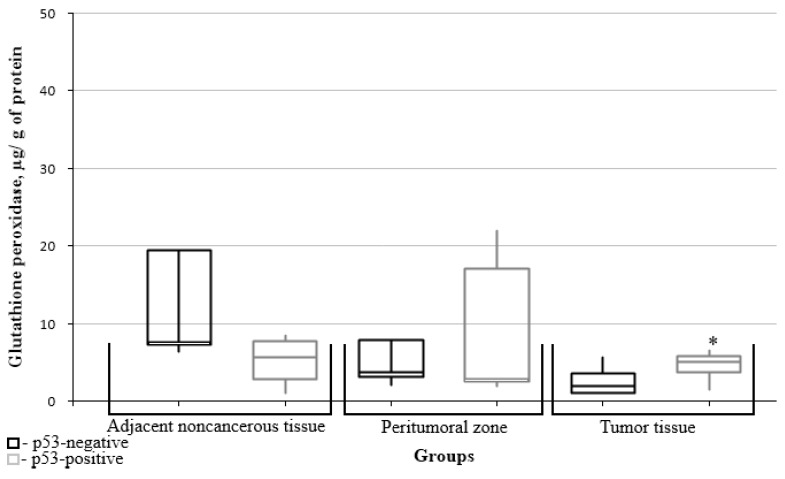
Medians and interquartile ranges (IQRs) of glutathione peroxidase levels in gliomal tissues as a function of the p53 protein tumor suppressor expression. There are significant differences of tumor glutathione peroxidase levels between the groups with/without expression of the p53 protein tumor suppressor (*—Mann-Whitney U-test *p* = 0.043).

The glutathione peroxidase content in tumor tissues was significantly higher in the p53 group (Figure 9).

**Figure 10:**
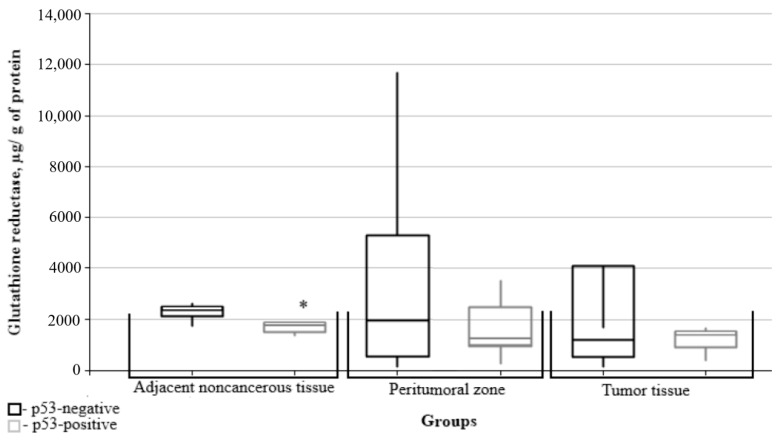
Medians and interquartile ranges (IQRs) of the glutathione reductase levels in gliomal tissues as a function of the p53 protein tumor suppressor expression. There are significant differences between glutathione reductase levels in noncancerous tissues adjacent to the tumors of groups with/without expression of the p53 protein tumor suppressor (*—Mann-Whitney U-test *p* = 0.043).

The glutathione reductase content in noncancerous tissues adjacent to the tumors was significantly lower in the p53 group (Figure 10).

Thus, it was found that the greatest number of glutathione system parameters depended on the value of the mitotic index Ki67, i.e., the levels of oxidized and reduced glutathione, glutathione peroxidase and glutathione transferase. Mutations of the isocitrate dehydrogenase IDH1 isoenzyme gene affected the content of reduced glutathione and glutathione peroxidase. Expression of the p53 protein tumor suppressor affected the content of glutathione peroxidase in the tumor tissue and of the glutathione reductase levels in adjacent noncancerous tissues. No significant differences were found between the indices of glutathione metabolism and of MGMT gene promoter methylation.

## 4. Discussion

It is now becoming clear that standard methods of radiation diagnosis and histological examination are often insufficient to correctly determine the prognostic potential of brain tumors.

Groupings of gliomas based on their malignancy grade are not homogeneous with respect to the aggressiveness of their behaviour and responses to treatment. Furthermore, isolated markers like Ki67, IDH, p53, and MGMT promoter methylation have limited predictive value.

Glutathione and the enzymes involved in its metabolism may, in some cases, be responsible for resistance to certain therapies. The study of glutathione levels in relation to other relevant immunohistochemical and molecular markers should help to identify early patterns of atypical gliomas and to find optimal treatment approaches.

The predictive role of glutathione level changes in relation to the treatment of gliomas has been extensively studied (https://www.ncbi.nlm.nih.gov/pmc/articles/PMC3608468/ (accessed on 6 September 2022), https://www.ncbi.nlm.nih.gov/pmc/articles/PMC6946871/ (accessed on 6 September 2022), https://www.ncbi.nlm.nih.gov/pmc/articles/PMC7821418/ (accessed on 6 September 2022), https://www.nature.com/articles/srep44792 (accessed on 6 September 2022)). Our study found an association between increased gliomal anaplasia and decreased levels of GT. Thus, an initially decreased level of GT in the peritumoral zone in Grade I and II gliomas may be a prognostically unfavourable factor in terms of a more rapid malignant transformation of such tumors. This would, therefore, justify more intensive therapy and more active dynamic monitoring of the patient. Based on these findings, it can be assumed that a decrease in its level in repeated operative interventions will also be a prognostic factor for the malignant transformation of tumors. These assumptions require further research.

The greatest number of significant differences and correlations that we found was between the parameters of glutathione metabolism (GSH, GSSG, GP, GST) and the value of the mitotic index Ki67. Since the content of this oncomarker demonstrates the extent of tissue proliferative activity, it may be assumed that tumor mitotic activity is largely related to the glutathione metabolism. The role of free-radical oxidation in carcinogenesis is well known [18]. Moreover, reactive oxygen species can both stimulate and inhibit carcinogenesis [19,20,21]. The glutathione metabolism system plays an important role in protecting cells from free-radical oxidation. According to our data, the level of oxidized and reduced glutathione decreases in the peritumoral zone tissues for high Ki67 mitotic indices.

Glutathione is a multifunctional molecule. It is an intracellular metabolism regulator. Its main role is in the GSH-antioxidant protection of cells. In addition to participating as a cofactor of glutathione peroxidase, GSH is able, non-enzymatically, to protect cells from free radicals, being a trap for them, thanks to the presence of its thiol group [22]. The role of GSH in apoptosis is well-established. The key mechanism for triggering apoptosis is the exit of GSH from cells. Cells, thus deprived of antioxidant protection, die [23]. GSH is known to play an important role in xenobiotics and in the metabolism of endogenous toxins [24]. It forms low-toxicity, easily excreted conjugates with drugs and other xenobiotics. In this regard, changes in the intracellular GSH content are factors in the development of many pathological conditions, including carcinogenesis [25]. The attachment of reduced glutathione to the thiol groups of proteins under the influence of glutathione transferase protects the SH-groups of these proteins from irreversible oxidation [26].

Glutathionylation controls the activity of AP1 and NFκB transcription factors, ubiquitin-dependent proteolytic protein degradation, cAMP, cAMP-dependent protein kinase, and other processes. It is assumed that the increased level of reduced glutathione and GT activity is consistent with observed increases in the S-glutathionylation processes [27,28,29]. Based on these facts and on the data, we have obtained, the degree of protection of proteins against proteolytic degradation at Grades III and IV appears significantly reduced. Since the Ki67 content demonstrates the expression of tissue proliferative activity, we can say that tumor mitotic activity is associated with a decrease of glutathione metabolism, especially in the peritumoral zone.

A significant decrease of glutathione peroxidase levels in the peritumoral zone tissues with high Ki67 mitotic indices, and the presence of a significant inverse correlation (Rho = −0.616) between these markers in the peritumoral zone demonstrates the importance of glutathione peroxidase for tumor proliferative activity.

The glutathione peroxidase family plays an important role in homeostatic control. Gpx1 is one of the most important free radical scavenging enzymes in the human body. Gpx1 oxidizes GSH (reduced form) to GSSG (oxidized form). The same enzyme may also reduce H_2_O_2_ to H_2_O. By changing the concentration of organic hydroperoxides, GPs are involved in controlling cell proliferation, cell survival, and apoptosis [30].

When both the mitotic index Ki67 and glutathione transferase levels were high, they were significantly lower in both the peritumoral zone and in the tumor tissue. Glutathione transferase detoxifies electrophilic xenobiotics. Thus, changes in the activity of this enzyme are related to multidrug resistance. It has been established that elevated levels of GSTs and glutathione are closely associated with the resistance of cancers to chemotherapeutic drugs [31]. However, the significant decrease that we detected of glutathione transferase levels in the peritumoral zone and in gliomal tumor tissue in the high mitotic index Ki67 group, and the observed inverse correlations between these parameters raise questions about the role of glutathione transferase in cell proliferation processes. Particularly, as the recent studies by Lei K., et al. (2020) [32] also indicate that GST1 (glutathione transferase 1) is associated with tumor cell proliferation and apoptosis.

In addition to their association with the Ki67 marker, our calculations revealed significant correlations of some glutathione system indicators with the IDH1 isoenzyme gene mutation as well. The interaction of the IDH1 marker with glutathione reductase, as shown using protein-protein interaction databases, was confirmed by significant correlation coefficients between these parameters in the peritumoral zone (Rho = 0.538). The bioinformatic analysis data were also confirmed, first, by the detected correlation between IDH1 and both the oxidized and reduced glutathione levels in the peritumoral zone and in tumor tissues (Table 3) and, second, by a significant increase of the reduced glutathione level in the group with mutation of the IDH isocitrate dehydrogenase enzyme gene. It is glutathione reductase as a reducing factor that restores GSSG using NADPH_2_ [33]. The wild-type IDH isoforms catalyze the oxidative decarboxylation of isocitrate to α-ketoglutarate, generating reduced NADPH_2_. The mutant IDH1 and IDH2 forms catalyze the conversion of α-ketoglutarate to oncometabolite 2-hydroxyglutarate with the formation of oxidized NADPH^+^ [34]. In the IDH1 mutation, α-ketoglutarate is converted to D-2-hydroxyglutarate, which, due to its structural similarity, acts as a competitive inhibitor that reduces the activity of α-KG-processing enzymes. As a feedback loop, α-ketoglutarate is replenished by glutaminolysis and the tricarboxylic acid cycle, resulting in lower glutamine and glutamate levels [35]. Consequently, IDHmut gliomas are “glutamate dependent,” and any glutamate deficiency reduces its metabolic interaction with cysteine. Lack of cytoplasmic cysteine reduces GSH synthesis, which increases susceptibility to reactive oxygen species stress. In this context, glutamate reduction contributes to better outcomes represented by gliomas with the IDH1 mutation [36]. Hence, gliomas with IDH1 mutation are characterized by slower tumor progression [37].

On the other hand, it is possible that IDH1 interacts with glutathione reductase at the level of NADPH_2_, which GSR uses to restore GSSG [3].

Glutathione peroxidase in association with glutathione reductase determines not only the nature of response to the effects of reactive oxygen species, but also the grade of tumor tissue pharmacoresistance [38].

The biologically significant interaction between IDH1 and glutathione peroxidase that we identified using protein-protein interaction databases was confirmed by the significant correlation coefficient between these parameters in the peritumoral zone (Rho = 0.570). In addition, there were significant differences in the levels of this enzyme in the peritumoral zone (*p* = 0.043) and in the groups with the wild-type or the IDH1 isocitrate dehydrogenase isoenzyme gene mutation. Gpx1 has been shown to regulate the sensitivity of glioma cell to oxidative stimuli [39]. In a study on cell cultures of gliomas with both the wild type and the IDH1 mutation, indirect evidence was found that glutathione peroxidase levels are altered in cells with the IDH1 mutation [40]. In addition, it was demonstrated that the glutathione peroxidase 1 expression dramatically increased with the IDH-wildtype [41].

Thus, the effects of the IDH1 mutation are mediated by glutathione levels and glutathione metabolism enzymes, this being most pronounced in the peritumoral zone tissues.

Bioinformatic analysis showed that the p53 protein can affect detoxification processes in different ways. It can control the level of expression of glutathione transferase or reduce MGMT expression. The significant inverse correlation that we found between the glutathione transferase level in adjacent noncancerous tissues and the expression of p53 (Rho = −0.606) is explained by the direct dependence of GSTP1 expression on p53 activity [42].

**Figure 11:**
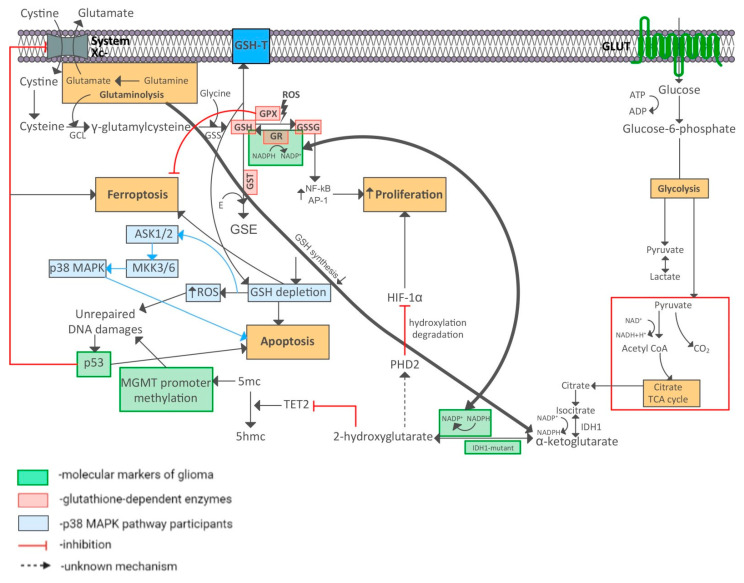
Diagram showing the relationships between gliomal molecular tumor markers and main enzymes involved in glutathione metabolism. GCL—γ-glutamyl-cysteine ligase; GSS—glutathione synthetase; GSH-T—glutathione transporter; system Xc-—cis-glu antiporter; E—electrophile; IDH—isocitrate dehyrogenase; GLUT—glucose transporter; PHD2—prolyl hydroxylase 2; HIF-1α—hypoxia-induced factor 1α; MGMT—O-6-methylguanine-DNA methyltransferase; ROS—reactive oxygen species; GSH—reduced glutathione; GSSG—oxidized glutathione; GST—glutathione S-transferase; GPx—glutathione peroxidase; GR—glutathione reductase; GSE—glutathione conjugate with electrophile; TET2—tet methylcytosine dioxygenase 2; 5mc—5-methylcytosine; 5hmc—5-hydroxymethylcytosine; ASK1/2—apoptosis signal-regulating kinase 1/2; MKK3/6—mitogen-activated protein kinase kinase 3/6.

A significant correlation was also found between MGMT gene methylation and the glutathione transferase levels in glial tumor tissue (Rho = 0.620). MGMT promoter methylation (no O-6-methylguanine-DNA methyltransferase enzyme activity) reduces the ability of tumor cells to repair damaged DNA sites after exposure to alkylating agent chemopreparations [43,44,45]. That is, the glutathione transferase content in a glial tumor increases when it is more sensitive to the effects of chemopreparations with alkylating agents. GST is also considered a major factor responsible for resistance to chemotherapy in gliomas or other malignancies. Thus, GST depletion increases the susceptibility of cancer cells to various forms of programmed cell death and their sensitivity to chemotherapy [2,46]. At the same time, elevated levels of glutathione, GSRs, and GSTs are closely associated with the resistance of cancers to chemotherapeutic drugs [31,38]. Some members of the GST superfamily have been found to play a role in cancer detoxification processes [29]. Some assumed relationship mechanisms between gliomal markers and enzymes involved in glutathione metabolism are shown in Figure 11. Our results suggest that the GST superfamily has many functions associated with molecular genetic changes in gliomas, and that its definitive role remains to be seen.

IDH1 isoenzyme gene mutations lead to oncometabolite 2-hydroxyglutarate accumulation instead of alpha-ketoglutarate. The former is involved in HIF-1α degradation and enhances tumor cell proliferation. α-ketoglutarate is replenished by glutaminolysis and the tricarboxylic acid cycle. This leads to glutamate depletion and to disruption of GSH synthesis. Excessive DNA damage accumulates, leading to apoptosis. Excessive transport of GSH into the extracellular matrix also leads to apoptosis [47].

2-hydroxyglutarate inhibits the activity of TET2 demethylase, which converts 5mc to 5hmc. Enzyme inhibition promotes gene silencing. MGMT gene silencing leads to a failure to eliminate DNA damage [48].

Tumor suppressor p53 suppresses the expression of gene SLC7A11, which encodes system Xc-. This increases the susceptibility of cells to ferroptosis due to cystine starvation. As cysteine (the reduced form of cystine) is a substrate for GSH synthesis, a deficiency of this amino acid restricts glutathione synthesis and GPX4 glutathione peroxidase activity, thereby initiating the ferroptosis (Stockwell, B. R., Jiang, X., & Gu, W. (2020). Emerging Mechanisms and Disease Relevance of Ferroptosis. Trends in Cell Biology. doi:10.1016/j.tcb.2020.02.009 (accessed on 19 September 2022); Seibt, T. M., Proneth, B., & Conrad, M. (2018). Role of GPX4 in ferroptosis and its pharmacological implication. Free Radical Biology and Medicine. doi:10.1016/j.freeradbiomed.2018 (accessed on 19 September 2022)).

The predominance of GSSG leads to the activation of NFkB and AP-1 stress-dependent transcription factors, thus enhancing cell proliferation. When glutathione reductase reduces oxidized glutathione, NADPH is oxidized. The reaction involving NADPH also occurs during α-ketoglutarate conversion into 2-hydroxyglutarate. Therefore, a cofactor-level relationship is possible.

## 5. Conclusions

Thus, it has been observed that the glutathione and the enzymes involved in its metabolism change their activity depending on the gliomal immunohistochemical profiles. The most pronounced relationship with glutathione metabolism is demonstrated by the levels of the nuclear protein Ki67 as a marker of proliferative activity and the presence of the IDH1 mutation as one of the key genetic events of gliomagenesis. The glutathione system is an active participant in the body’s antioxidant defense, involving p53 markers and MGMT promoter methylation and allowing the characterization of the state of the gliomal cells at different stages of tumor development.

## Figures and Tables

**Table 1:** Morphometric evaluation of tumor markers in gliomal tissue of different anaplasia grades in 20 individuals.

Grade	I	II	III	IV
Number of patients per group	1	6	3	10
IDH1—% of mutation detection	0%	100%	100%	30%
MGMT—% of methylation detection	100%	83%	100%	30%
p53—% of detection	0%	66%	0%	80%
High Ki67 (>10%)—% of detection	0%	0%	100%	100%
Low Ki67 (<10%)—% of detection	100%	100%	0%	0%
Ki67 median	-	2%	12%	37%
Ki67 interquartile intervals (25–75)	-	1.5–3%	-	25–40%

**Table 2:** Indicators of glutathione metabolism in gliomas.

Glutathione Metabolism Parameter		Adjacent Noncancerous Tissues (Median; Quartiles)	Peritumoral Zone		Tumor	
			Median; Quartiles	Mann-Whitney U-Test	Median; Quartiles	Mann-Whitney U-Test
Oxidized glutathione, μmol/l	Grade I (*n* = 1)	12,583.00	13,636.00		10,532.00	
	Grade II, (*n* = 6)	11,514.50 (10,897.465– 12,141.75)	14,359.00 (13,350.75–15,753.50)	* *p* = 0.054	11,430.50 (9772.00–12,059.25)	*p* = 0.631
	Grade III, (*n* = 3)	12,256.00 (11,191.00– 12,661.04)	8137.00 (8124.79 9019.50)	* *p* = 0.049	4013.00 (3635.89 4975.50)	* *p* = 0.050
	Grade IV, (*n* = 10)	11,363.33 (11,146.00– 11,853.17)	9021.00 (8462.97–9837.00)	* *p* = 0.007	6854.65 (6464.75–7006.58)	* *p* = 0.004
Reduced glutathione, μmol/l	Grade I (*n* = 1)	11,239.00	15,210.00		1323.00	
	Grade II, (*n* = 6)	9342.00 (8783.75–9922.75)	10,639.50 (7043.55–11,928.00)	*p* = 0.522	14,132.00 (13,294.81–14,675.25)	* *p* = 0.003
	Grade III, (*n* = 3)	10,396.00 (10,033.00–11,347.78)	6921.00 (5174.08–7725.50)	* *p* = 0.049	7813.00 (7059.00–8641.78)	* *p* = 0.049
	Grade IV, (*n* = 10)	9705.62 (9417.06– 9970.00)	8004.50 (6478.75–8315.22)	*p* = 0.109	8006.50 (7777.00–8636.50)	* *p* = 0.054
Glutathione peroxidase, µg/g of protein	Grade I (*n* = 1)	8.25	24.71		21.30	
	Grade II, (*n* = 6)	6.84 (5.89–7.44)	20.00 (19.25–21.50)	* *p* = 0.004	3.79 (3.52–4.73)	* *p* = 0.010
	Grade III, (*n* = 3)	6.43 (4.33–7.06)	3.88 (2.89–4.07)	*p* = 0.275	3.78 (2.43–5.12)	*p* = 0.513
	Grade IV, (*n* = 10)	7.72 (7.06–12.89)	3.22 (2.56–4.37)	* *p* = 0.039	2.63 (1.41–3.78)	* *p* = 0.0002
Glutathione reductase, µg/g of protein	Grade I (*n* = 1)	2105.00	3179.00		2769.23	
	Grade II, (*n* = 6)	1802.00 (1625.25–1872.17)	3188.50 (3029.00–3452.25)	* *p* = 0.007	1536.49 (1351.56–1698.41)	*p* = 0.149
	Grade III, (*n* = 3)	1750.28 (1657.14–1810.64	973.00 (569.84–987.00)	* *p* = 0.049	1134.00 (895.02–1338.36)	* *p* = 0.049
	Grade IV, (*n* = 10)	1894.00 (1482.49–2252.00)	994.35 (685.42–1739.20)	*p* = 0.065	907.79 (420.39– 1374.25)	* *p* = 0.011
Glutathione transferase, µg/g of protein	Grade I (*n* = 1)	28.25	78.19		67.31	
	Grade II, (*n* = 6)	20.07 (16.60–22.08)	70.70 (66.60 –74.61)	* *p* = 0.004	54.47 (41.82–61.49)	* *p* = 0.007
	Grade III, (*n* = 3)	27.00 (26.02–28.50)	19.00 (18.52–21.00)	* *p* = 0.005	15.00 (13.48–24.17)	*p* = 0.513
	Grade IV, (*n* = 10)	35.17 (32.89–55.20)	33.24 (23.21–40.51)	*p* = 0.064	12.09 (7.37–16.32)	* *p* = 0.001

Legend: *—statistically significant differences in comparison with adjacent noncancerous brain tissues.

**Table 3:** Correlations (Spearman’s coefficient) between the immunohistochemical markers of gliomas and the parameters of glutathione metabolism of brain tumors in 20 individuals.

Parameter of Glutathione Metabolism		Markers of Gliomas			
		IDH	Ki67	MGMT	p53
Oxidized glutathione Rho (p)	Adjacent noncancerous tissue	0.598 (0.052) *	−0.260 (0.392)	0.169 (0.689)	−0.173 (0.656)
	Peritumoral zone	0.570 (0.086)	−0.534 (0.060)	0.169 (0.689)	0.087 (0.825)
	Tumor tissue	0.597 (0.051) *	−0.674 (0.012) *	0.169 (0.689)	0.260 (0.500)
Reduced glutathione Rho (p)	Adjacent noncancerous tissue	0.239 (0.479)	−0.273 (0.391)	0.056 (0.895)	−0.346 (0.361)
	Peritumoral zone	0.359 (0.279)	−0.550 (0.051) *	0.056 (0.,895)	0.327 (0.429)
	Tumor tissue	0.837 (0.001) *	−0.765 (0.002) *	0.169 (0.689)	−0.173 (0.656)
Glutathione peroxidase Rho (p)	Adjacent noncancerous tissue	−0.210 (0.536)	0.359 (0.252)	−0.510 (0.197)	−0.433 (0.244)
	Peritumoral zone	0.570 (0.049) *	−0.616 (0.025) *	0.056 (0.895)	−0.087 (0.825)
	Tumor tissue	−0.179 (0.598)	−0.282 (0.351)	0.394.(0.334)	0.519 (0.051) *
Glutathione reductase Rho (p)	Adjacent noncancerous tissue	−0.119 (0.726)	0.166 (0.588)	−0.056 (0.895)	−0.606 (0.043) *
	Peritumoral zone	0.538 (0.048) *	−0.338 (0.283)	−0.507 (0.199)	0.001 (0.999)
	Tumor tissue	−0.179 (0.598)	−0.268 (0.376)	0.394.(0.334)	−0.086 (0.824)
Glutathione transferase Rho (p)	Adjacent noncancerous tissue	−0.120 (0.726)	0.663 (0.014) *	−0.158 (0.735)	−0.606 (0.043) *
	Peritumoral zone	0.359 (0.279)	−0.531 (0.062)	−0.282 (0.244)	0.001 (0.999)
	Tumor tissue	0.119 (0.726)	−0.597 (0.031) *	0.620 (0.012) *	−0.087 (0.825)

Legend: Statistically significant differences are reported * *p* < 0.05.

## Data Availability

The datasets generated during and/or analyzed during the current study are available from the corresponding author on reasonable request.

## Supplementary Materials

The following supporting information can be downloaded at: https://www.mdpi.com/article/10.3390/biomedicines10102393/s1, Table S1: Clinicopathologic features of gliomas patients
