# Supplementary material for: Relationship between Glutathione-Dependent Enzymes and the Immunohistochemical Profile of Glial Neoplasms
Source: Biomedicines. 2022 Sep 25;10(10):2393. doi: 10.3390/biomedicines10102393 (PMC9598304; doi:10.3390/biomedicines10102393)
Supplement: Supplementary file 1 [file biomedicines-10-02393-s001.zip › biomedicines-1877717-SI.pdf]

Supplementary information

Additional file 1 of « RELATIONSHIP OF GLUTATHIONE-DEPENDENT ENZYMES AND THE IMMUNOHISTOCHEMICAL PROFILE OF GLIAL NEOPLASMS»

Supplementary Table S1. Clinicopathologic features of gliomas patients

Characteristics	Grade I (n=1)	Grade II (n=6)	Grade III (n=3)	Grade IV (n=10)
Ages (years)				
< 60	1	2		3
≥ 60		4		7
Gender				
Male		2		7
Female	1	4		3
Average tumor volume (cm/^{3})	121,9	99,06	66.6	89,7
